# Biomechanical Properties of the Aortic Wall: Changes during Vascular Calcification

**DOI:** 10.3390/biomedicines11010211

**Published:** 2023-01-14

**Authors:** Jinwen Zhou, Manasa Reddy Gummi, Anna Greco, Milen Babic, Jaqueline Herrmann, Farid I. Kandil, Markus van der Giet, Markus Tölle, Mirjam Schuchardt

**Affiliations:** 1 Charité-Universitätsmedizin Berlin, Corporate Member of Freie Universität Berlin and Humboldt Universität zu Berlin, Department of Nephrology and Medical Intensive Care, Hindenburgdamm 30, 12203 Berlin, Germany; 2 Charité-Universitätsmedizin Berlin, Corporate Member of Freie Universität Berlin and Humboldt Universität zu Berlin, Institute of Social Medicine, Epidemiology and Health Economics, Luisenstraße 57, 10117 Berlin, Germany; 3 Charité-Universitätsmedizin Berlin, Corporate Member of Freie Universität Berlin and Humboldt Universität zu Berlin, Department of Pediatric Oncology/Hematology, Otto-Heubner Centre for Pediatric and Adolescent Medicine, Augustenburger Platz 1, 13353 Berlin, Germany

**Keywords:** aorta, biomechanical properties, calcification, passive response, stress, stretch

## Abstract

Medial vascular calcification (MAC) is characterized by the deposition of hydroxyapatite (HAP) in the medial layer of the vessel wall, leading to disruption of vessel integrity and vascular stiffness. Because currently no direct therapeutic interventions for MAC are available, studying the MAC pathogenesis is of high research interest. Several methods exist to measure and describe the pathophysiological processes in the vessel wall, such as histological staining and gene expression. However, no method describing the physiological properties of the arterial wall is currently available. This study aims to close that gap and validate a method to measure the biomechanical properties of the arterial wall during vascular calcification. Therefore, a stress–stretch curve is monitored using small-vessel-myography upon ex vivo calcification of rat aortic tissue. The measurement of biomechanical properties could help to gain further insights into vessel integrity during calcification progression.

## 1. Introduction

Cardiovascular diseases (CVD) lead to the highest number of deaths worldwide [1]. The cardiovascular morbidity and mortality rate is higher in patients with chronic kidney disease (CKD) compared to patients with normal kidney function. Medial arterial calcification (MAC) is common in CKD patients and is associated with high CVD risk [2]. The main drivers of MAC in CKD patients include aging, disturbance of calcium and/or phosphate metabolism, inflammation, and uremic toxins [2,3]. MAC is characterized by inappropriate deposition of calcium phosphate salts in the form of hydroxyapatite (HAP) within the vascular wall and results in damage of the vessel structure [2]. The structural integrity of the vessel wall provides mechanical homeostasis for normal function. The arterial wall consists of three distinct layers: the tunica intima, the tunica media, and the tunica adventitia. The structural components and the wall thickness are different between large, medium, and small arteries. In general, the innermost layer is composed of endothelial cells covering the luminal surface of the blood vessel. The tunica media comprises circumferentially aligned elastin and collagen fibers, as well as vascular smooth muscle cells (VSMC). The outer layer consists of fibroblasts, elastin and mainly collagen fibers, which are oriented longitudinally aligned as wavy bundles [4].

Vascular remodeling accompanying MAC is critical for mechanical homeostasis and the development of CVD [2,3,5]. To investigate the mechanisms of the MAC progression, many models have been established, especially in the past few decades [6]. Most protocols focus on the analysis of the calcified areas, including quantification of the calcification level and changes in expression patterns of several involved proteins. However, studying the biomechanical properties of the calcified tissue may provide further insights into vascular calcification progression, because the active biological process of MAC is highly associated with elastin degradation, excessive collagen deposition and glycosylation, which pathophysiologically modifies the vessel wall structure [2,3,5]. As ex vivo settings with aortic tissue preserve the vessel structure and tissue integrity, it provides the possibility to study stresses and strains calculated by forces and loaded dimensions.

The current study aims to establish and validate a method to investigate these biomechanical properties in the process of vascular calcification using an ex vivo setting. For the induction of vascular calcification, two inducers known from in vitro and in vivo settings were used: high phosphate medium and azathioprine (AZA) [7,8]. The results clearly demonstrate that the biomechanical properties were distinguishable between control and stimulation, and therefore provide novel markers to describe vessel functionality during calcification progression.

## 2. Materials and Methods

**Figure 1:**
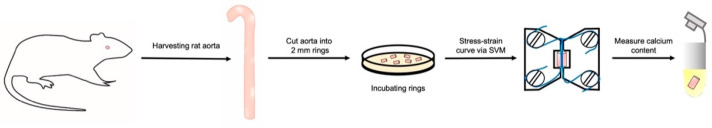
Scheme of the workflow. SVM: small-vessel myography.

The complete and stepwise workflow of the experimental procedures is summarized in Figure 1.

### 2.1. Aorta Harvest

Male Wistar rats were purchased from Janvier Labs. Animals with an average age of 1–3 months (mean weight 434 ± 77 g) were used for this study. The animals were kept in cages under standard conditions and fed a normal chow diet with water ad libitum (temperature: 23 ± 1 °C, humidity: 45 ± 10%). For aortic removal, anesthesia was induced by intraperitoneal injection of pentobarbital (400 mg/kg body weight). After reaching and checking for anesthesia depth, the thoracic and abdominal cavities were opened, the lungs and heart were removed, and the aorta was separated from the heart to the peritoneum. The aorta was transferred to a cell culture dish with phosphate-buffered saline (PBS) (Biochrom AG (part of Merck Millipore, Darmstadt, Germany). Fat tissue surrounding the aorta was carefully removed under the stereomicroscope with micro-scissors and forceps without impairment of the tissue.

### 2.2. Ex Vivo Stimulation of Aortic Rings

The thoracic aorta (without the aortic arch) was cut into rings about 2 mm in length and the rings were numbered from proximal to distal. The rings were stimulated ex vivo as indicated and afterwards harvested to measure the passive response.

All cell culture components were obtained from Bio&Sell (Feucht, Germany) and Biochrom AG (part of Merck Millipore, Darmstadt, Germany). As a stimulation medium, Dulbecco’s-modified Eagle medium (DMEM) supplemented with 15% fetal calf serum (FCS) and 1% penicillin/streptomycin was used as a control medium (COM). The calcifying medium (CAM) additionally contains 5 mmol/L sodium dihydrogen phosphate and 284 µmol/L ascorbic acid. The stimulation times vary from 0 (basal), 3, 7, 14, to 21 days as indicated. The stimulation medium was changed every two to three days.

### 2.3. Passive Response

Upon indicated stimulation, vessel segments of the 2 mm aortic rings were mounted between two jaws into the small vessel myograph (model 610; Danish Myo-Technology A/S, Hinnerup, Denmark) and maintained at 37 °C in PBS with constant carbogen bubbling. The rings were initially in a completely unloaded state. For the experiment, the distance between the tungsten wire vessel holders (Δ) was elongated in 0.05 mm steps by manual adjustment of the micrometer until it reached failure. Both the displacements between the vessel holders (Δ) and the tensile force (F) were recorded. After the test, the ring was cut, and the circumference (C) was measured with a ruler. The internal diameter of the ring was calculated as (d) = circumference(C)/π. The diameter of the vessel holder (e_0_) was 0.04 mm, according to the manual [9]. The thickness of the ring (e_0_) was referenced in a previous article e_0_ ≈ 0.08967 mm among 0–3-months aged rats [10]. The width of the ring (a_0_) was about 2 mm.

The stress–strain curve is characterized by three equations [11,12].

(1) $$\sigma =\frac{F}{2a_{0}e_{0}}\lambda$$

Equation (1). Cauchy stress (σ). F: force; λ: stretch; a_0_: ring width; e_0_: ring thickness

(2) $$\lambda =1+2\frac{\left(\Delta -\Delta_{0}\right)}{\pi d}$$

Equation (2). Stretch (λ). d: ring diameter; Δ_0_: initial length where the ring starts to resist deformation; Δ − Δ_0_: the elongation in the separation of the wires by manual adjustment of the micrometer every time.

(3) $$\Delta_{0}=\frac{\pi }{2}\left(d-\phi \right)$$

Equation (3). Initial length (Δ_0_). Δ_0_: initial length where the ring starts to resist deformation; d: ring diameter; ϕ: vessel holder diameter

**Figure 2:**
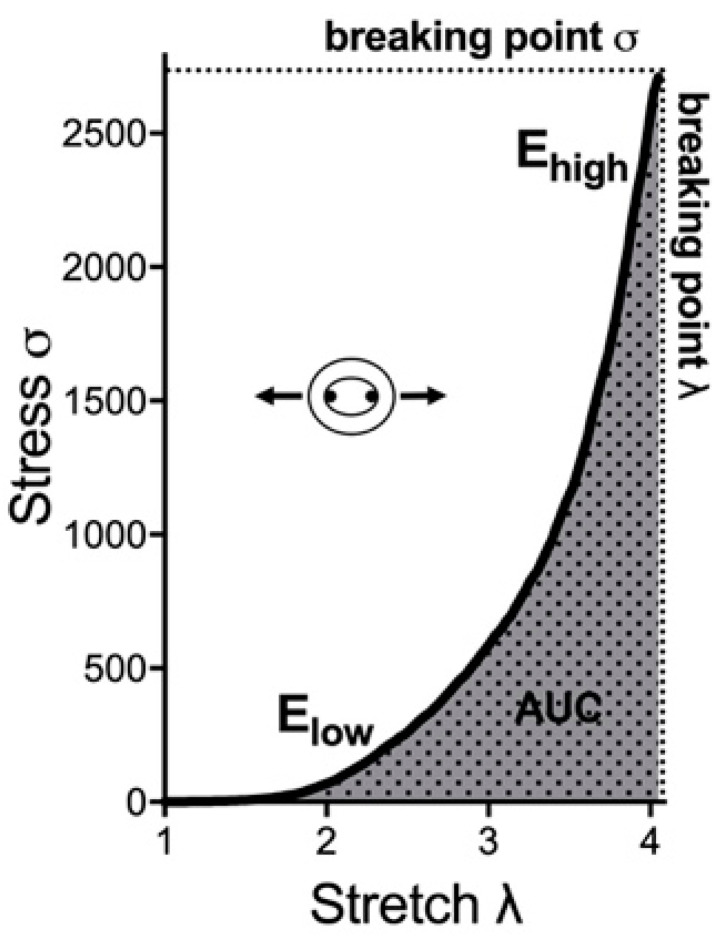
Exemplary stress–stretch curve and respective parameters for biomechanical properties. E_low_ and E_high_ are the modulus of the first and last 10% of the curve, respectively. AUC is the area under the stress–stretch curve. The breaking point is the point of the ring fracture. AUC: area under the curve.

The stresses and strains of the aortic tissue are calculated by forces and loaded dimensions of the aortic tissue. Five biomechanical parameters out of the stress–stretch curve were analyzed to determine the passive mechanical response of the arterial wall (Figure 2). The incremental elastic modulus (E) of the first and last 10% strain of the stress–stretch curve was calculated as E_low_ and E_high_. E_low_ represents the contribution of the elastin fibers to stiffness. E_high_ represents the contribution of the collagen fibers to stiffness. The area under the curve (AUC) of the stress–stretch curve represents the energy absorbed by the ring. The breaking point, including breaking point-stress (σ) and breaking point-stretch (λ), represents the maximum stress until the ring fracture (Figure 2).

### 2.4. Calcium Content

The aortic rings were decalcified in hydrochloric acid (0.6 mmol/L). Calcium content was quantified using the colorimetrical o-cresolphthalein method (Colorimetric Calcium Assay, Sciencell^TM^, Berlin, Germany) according to the manufacturer’s protocol. The determined absolute calcium content was normalized to the dry weight of the respective aortic ring.

### 2.5. Gene Expression

Cryo-conserved aortic tissue was homogenized using the Retsch mixer mill (MM 301). Total RNA was isolated and purified using Trizol^®^ (Fisher Scientific, Langenselbold, Germany) and cDNA was obtained by reverse transcription using the iScript cDNA synthesis kit (BioRad, Munich, Germany) according to the manufacturer’s protocol. Quantitative determination of mRNA expression was conducted using the iQ™ SYBR Green supermix and the CFX384 real-time PCR detection system (CFX software version 3.1, BioRad, Munich, Germany). Reactions for each sample were performed in technical duplicates. The primer pairs for matrix metalloproteinase 9 (*Mmp-9*), collagen type III (*Col3a1*), bone morphogenetic protein 2 (*Bmp-2*), and ribosomal protein L13a (*Rlp13a*) were purchased from Biomol (Hamburg, Germany). Relative expression values were evaluated with the 2^−ΔΔCt^ method and values were normalized to *Rpl13a* housekeeper gene expression.

### 2.6. Statistical Analysis

A minimum of three independent experiments were conducted to confirm the reproducibility of the results. Data are presented as mean ± standard error of mean, unless otherwise indicated. The statistical significance between treatment groups was determined using ANOVA and a t-test using the GraphPad Prism software 5.0 (GraphPad Software Inc., version 9, San Diego, USA) and custom-written code for Python. A *p*-value of <0.05 was considered statistically significant. For further comparison, effect sizes are given.

## 3. Results

In all experimental sets, the calcium content of the tissue as current standard assay for calcium quantification of the tissue was also measured.

### 3.1. Biomechanical Properties of Rat Aortic Tissue: Proof-of-Concept

The first set of experiments investigated the basal (w/o stimulation) biomechanical properties in different regions of the aorta from male rats. Subsequently, the thoracic aorta was cut into 18 rings of ~2 mm length and the stress–stretch curve was determined. All detected parameters such as E_low_, E_high_, AUC, breaking point (σ), breaking point (λ), and calcium content did not differ significantly between different parts of the thoracic aorta under basal conditions (Supplementary Table S1). Furthermore, the biomechanical aortic wall properties were analyzed upon ex vivo stimulation of the aortic tissue with CAM for 14 days. Again, the detected parameters did not differ significantly between the aortic rings from different aortic locations (Supplementary Table S2).

However, as expected, a significant induction of vascular calcification, detected by the tissue calcium content, could be determined by comparing CAM stimulation for 14 d vs. basal (w/o stimulation) (Table 1). This was accompanied by changes in the biomechanical properties of the aortic tissue, as shown by the significant changes in E_low_, E_high_ and both breaking points (Table 1). The effect size of the changes was higher compared with the current “gold standard” of tissue calcium content.

In the following stimulation experiments, the aorta from one animal was cut into several parts to determine the time-dependent effects of changes in biomechanical properties upon vessel wall calcification and thereby address the 3R principle for the reduction of animal material [13].

### 3.2. Biomechanical Properties: COM vs. CAM

**Figure 3:**
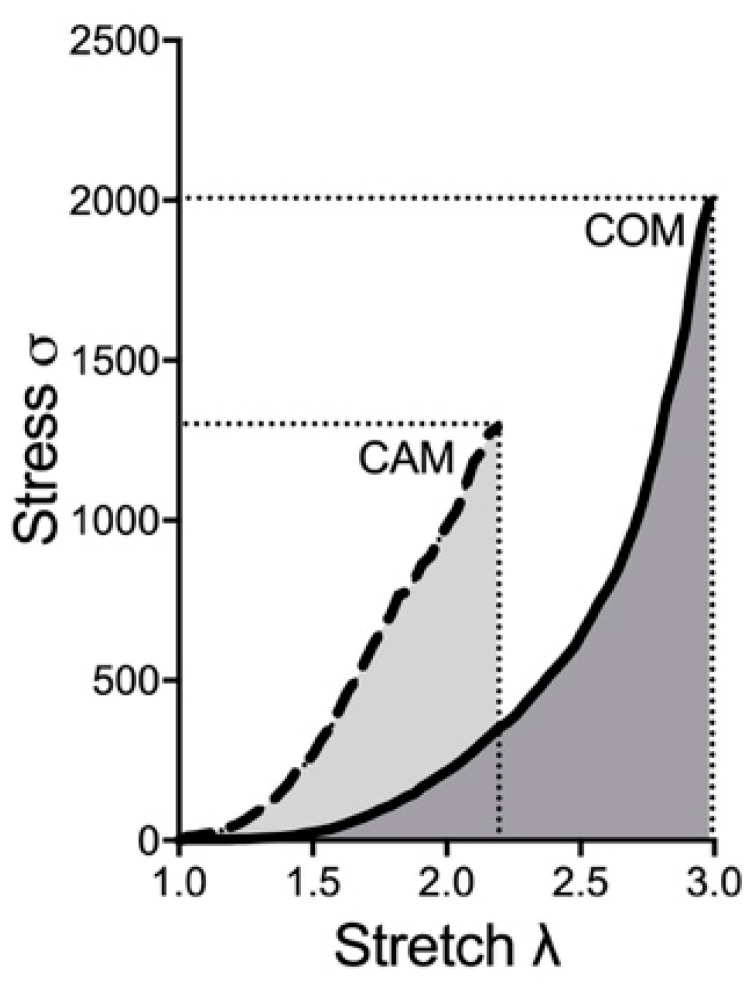
**Exemplary stress–stretch curve upon COM and CAM treatment**. Aortic rings were incubated in COM or CAM respectively for 14 d. Stress–stretch curve was determined via small-vessel myography. COM: control medium, CAM: calcification medium.

In the next step, the biomechanical properties were determined under conditions currently used in ex vivo calcification experiments. Therefore, the tissue was incubated for 14 days using COM and CAM [6]. As shown as an example in Figure 3, the stress–stretch curve of the aorta shifts, leading to changes in the biomechanical properties and mentioned parameters.

**Figure 4:**
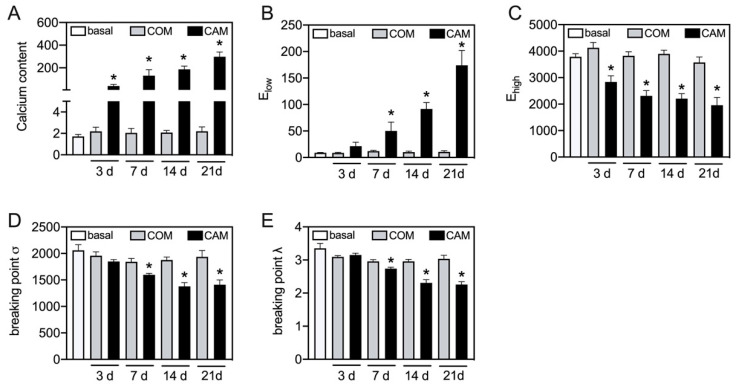
**Time-dependent changes in stress–stretch curve parameters**. Aortic tissue rings were incubated for indicated time points with COM and CAM. The stress–stretch curve and respective parameters were determined. Calcium content (µg/mg) was quantified upon decalcification of the tissue. n > 3, * *p* < 0.05 compared to COM at respective time points. COM: control medium, CAM: calcification medium.

Therefore, the time-dependent effect of changes in the biomechanical properties should be elucidated. The aortic tissue was incubated ex vivo with COM or CAM for 0, 3, 7, 14, and 21 days. The chosen time points were in common with previous studies. While no significant effect on detected parameters was seen in COM-stimulated tissue, CAM stimulation leads to an induction of vascular calcification and corresponding changes in the stress–stretch curve (Figure 4). The calcium content increases over time and significantly starts to differ at 3 d of incubation (Figure 4A). In addition, a similar trend was seen for E_low_, which time-dependently and significantly increases upon 7 d of incubation in CAM (Figure 4B). E_high_ decreases over time, while the effect becomes significant upon 3 d of incubation in CAM (Figure 4C). The breaking points (stress and stretch) significantly decrease upon 7 d of incubation in CAM (Figure 4D,E).

### 3.3. Changes in Vascular Elastin and Collagen

**Figure 5:**
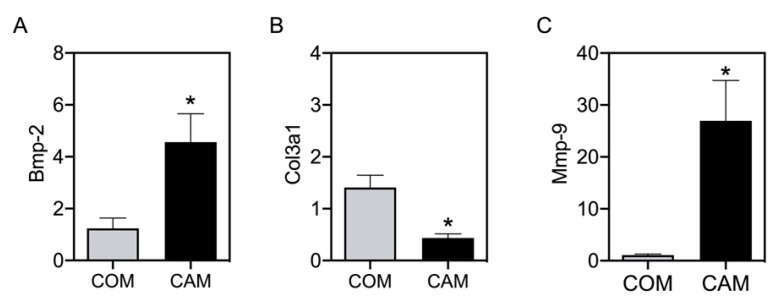
**mRNA expression in aortic tissue**. Aortic tissue rings were incubated for 14 d with COM and CAM. mRNA gene expression was determined via real-time polymerase chain reaction. n ≥ 5, * *p* < 0.05 compared to COM. COM: control medium, CAM: calcification medium.

The values of E_low_ and E_high_ are predominantly representative of the elastin and collagen to stiffness, respectively [14]. Therefore, we next tested whether detected changes in E_low_ and E_high_ correspond to changes in aortic gene expression. Bmp-2 was measured as an osteogenic marker. As expected, and as is known from previous studies, Bmp-2 significantly increases in CAM-treated aortic tissue (Figure 5A). In addition, Col3a1 significantly decreases in CAM-treated aortic tissue compared to its respective COM-treated tissue (Figure 5B). Mmp-9, as one matrix metalloproteinase cleaving elastin, was significantly increased upon CAM stimulation (Figure 5C).

### 3.4. Method Validation by a Known Inductor of Vascular Calcification: Azathioprine

**Figure 6:**
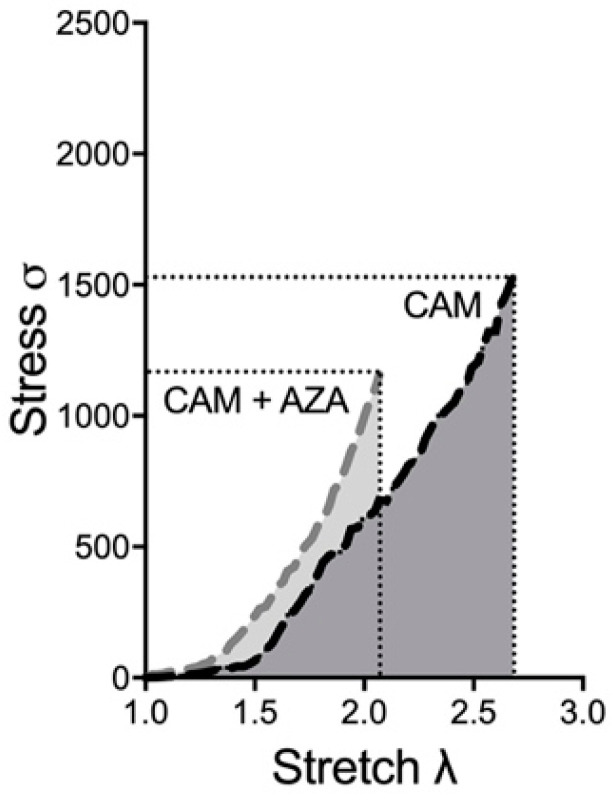
**Exemplary stress–stretch curve upon CAM and CAM + AZA treatment**. Aortic rings were incubated in CAM and CAM + AZA respectively for 14 d. Stress–stretch curve was determined via small-vessel myography. CAM: calcification medium, AZA: azathioprine.

To confirm whether vascular calcification changes the investigated biomechanical properties of the arterial wall, we also tested AZA, which is known to induce vascular calcification from previous in vitro, ex vivo and in vivo studies [7,8]. Here, we wanted to analyze whether AZA treatment further changes the biomechanical properties of the aortic wall. Figure 6 depicts the exemplary shift in the stress–stretch curve upon CAM + AZA treatment, compared to CAM stimulation alone. Therefore, here again the time-dependent effect was elucidated.

**Figure 7:**
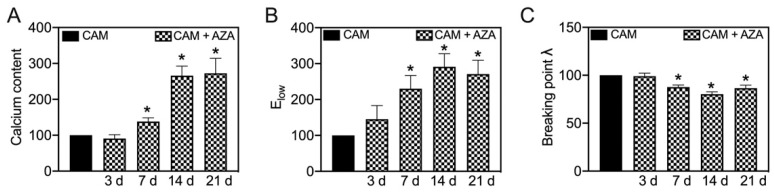
**Effect of azathioprine on biomechanical properties under calcifying conditions.** Aortic tissue rings were incubated for indicated time-points with CAM and AZA (100 µmol/L) and the stress–stretch curve and respective parameters were determined. Calcium content was determined upon decalcification of the tissue. Values are normalized to CAM stimulation at respective time points. n = 6, * *p* < 0.05 compared to CAM at respective time points. CAM: calcification medium, AZA: azathioprine.

In line with our previous study [7], AZA treatment further increases the calcification of aortic tissue in a time-dependent manner (Figure 7A). This also leads to changes in E_low_ and the breaking point stretch, which significantly increase (Figure 7B) or respectively decrease (Figure 7C) upon 7, 14 and 21 d of AZA treatment. While E_high_ and the breaking point stress did not change significantly upon AZA treatment, the AUC significantly decreases upon 7 and 14 d of treatment compared to its respective controls (Supplementary Figure S1).

## 4. Discussion

The results demonstrate that the investigation of biomechanical vessel wall properties could expand the analyzing tools in the research field of vascular calcification. The stress–stretch curve changes upon induction of calcification using CAM and AZA in a time-dependent manner.

The complex structural and functional composition of the vessel wall is a prerequisite for maintaining its normal function with unique mechanical properties [15]. Under physiological conditions, the blood vessel wall is built to withstand and propagate the forces applied by the blood flow and the surrounding tissues. Pressure induces internal circumferential or hoop stress in the vessel wall. Similarly, internal longitudinal stress is induced due to the distending force in the longitudinal direction. The surrounding tissue at the ends and at several locations along its length produces a second tensile force, which is responsible for the longitudinal stress due to tethering. The blood flow induces shear stress tangential to the flow axis. During vessel calcification, deposition of HAP in the medial vessel wall leads to vascular remodeling, damaging the microstructural composition and therefore inducing changes in the forces including the biomechanical properties. Any disturbance of these properties can significantly compromise vessel function. The characterization of the biomechanical properties is essential to better understand the progression of vascular calcification.

Arterial wall biomechanical properties are nonlinear and vary with the direction of deformation and loading. Nonlinear stress–strain curves can be constructed from force and loaded dimension in ex vivo experiments [12,16,17,18,19,20]. Conventional mechanical tests for vessels include, but are not limited to tensile, tensile stress-relaxation, compression, burst pressure and compliance dynamic mechanical analysis. The main differences result from the type of applied load and deformation. This can be compressive or tensile, incremental or cyclic, uniaxial or pressure-based. In the present study, mechanical tensile tests using small-vessel myography were conducted to confirm changes in biomechanical properties during MAC progression in an ex vivo setting.

The current study investigates thoracic aortas in rats. It is already known that stress–strain curves are different between the thoracic and abdominal parts of the aorta [12]. More branching points in the abdominal aorta compared to the thoracic aorta may contribute to increased abdominal calcification. In branching points, the blood flow is more turbulent and non-uniform, and irregular distribution of shear stress impairs endothelial function. This is in line with another study showing higher calcium deposition in the abdominal aorta compared to its thoracic part [21]. The current study aims to investigate the biomechanical properties of the thoracic aortic wall during calcification progression in a time-dependent manner and compares it to the current “gold standard” of calcium quantification of the aortic tissue. While the calcium content quantifies the amount of calcium deposits, the tensile testing describes the disruption of physiological properties during calcification progression. Therefore, this test may expand the analyzing tools in calcification research.

The first part of this study showed that the biomechanical properties and calcium content are uniform in different areas of the thoracic aorta of the rats at basal level (0 days w/o stimulation) and upon 14 d of CAM (high phosphate medium) incubation. While incubation of the aortic tissue in the standard culture medium (COM) did not alter the biomechanical properties of the aortic tissue time-dependently, incubation in CAM with a high phosphate condition leads to time-dependent changes in the stress–stretch curve. Because the tensile testing is not disruptive for the tissue, the calcium quantification can also be performed afterwards.

To further validate the current stress−stretch curve setting, we also tested the effects of AZA on biomechanical parameters. For AZA we have already demonstrated that the production of reactive oxygen species and inflammation results in vascular calcification in in vitro and ex vivo models [7,8]. Our in vivo study revealed an increase in pro-inflammatory cytokines in AZA-treated rats compared to controls [8]. From other studies, it is known that pro-inflammatory cytokines promote arterial remodeling and vascular calcification [22]. In line with the previous results, AZA affects the vascular integrity of the vessel wall and significantly changes biomechanical properties. Here, especially E_low_ and breaking point λ are of the greatest interest. The four mentioned biomechanical properties represent different aspects of vessel integrity. Here, E_low_ appears to discriminate better for changes in elastic modulus than E_high_. In the condition of structural vessel abnormalities, ring fractures occurred at a lower stretch associated with a higher variation especially in the parts of the higher stretch. For the breaking points, λ appears to have a higher sensitivity to changes in the elastic modulus compared to σ as the maximum stress leading to ring rupture is reached by a lower stretch. Thus, E_low_ and the breaking point λ could be valuable parameters for early detection of structural changes related to vascular calcification.

While the breaking points determine tissue rupture, the value E_low_ of the stress−stretch curve represents the elastin contribution to stiffness, whose increase represents elastin destruction or degradation in the process of vascular calcification. Elastin is an elastic protein polymer with a helical structure and an important component of elastic fibers [23]. Elastin is abundant in elastic tissue as the aorta [24]. Aortic elasticity is necessary for proper cardiovascular function. Elastin loss or disruption of elastic fibers is associated with stiffening [25,26]. The disorganization or insufficiency and improper assembly, fragmentation, and biochemical modifications of elastin are associated with many cardiovascular diseases [27,28,29]. Elastic fibers can calcify due to the presence of mineralization nucleation sites including sulfhydryl and carboxyl groups, matrix glycoproteins, glycosaminoglycans, lipoproteins, and calcium-binding proteins such as matrix Gla protein [30,31,32,33,34,35]. However, the molecular mechanism and the specific role of elastin in vascular calcification progression have not been fully elucidated. The elastin content seems to be critical in MAC; however, the mechanisms are currently not well understood [27]. Elastin is synthesized and secreted by medial-located VSMC of the arterial wall [24,27]. Reduced elastin amounts are associated with increased stiffness and altered vessel structure influencing the mechanical and functional properties of the vessel [12,27,36]. While elastin-null mice die within a few days after birth, heterogeneous deletion of elastin in mice leads to a normal life span with a decreased elastin amount of 50–60% [27]. Arteries from these mice show reduced stretch and increased length [27]. This seems in line with the reduction in breaking points found in the calcified vessels (stimulated with CAM) compared to control vessels (stimulated with COM). Mice with a fibrillin-1 deficiency gene have severe calcification in aortic media due to the disruption of the microfibrillar assembly and dysfunction of elastin [28,37]. The initial phases of calcification appeared to be related to elastin fragments. Elastin comprises some uncharged or neutral binding sites, which easily bind to positive calcium ions. In a physiological situation, elastin protein is surrounded by glycoproteins such as fibulin and fibrillin to prevent binding to calcium ions. Due to elastin degradation in the pathological situation, elastin loses these coats and becomes amorphous to calcium-binding. Elastin, being positively charged due to the bound calcium ions, attracts negative-charge carbonate and phosphate ions, and can become the initial nidus. Constant elastin degradation further aggravates vascular calcification [30,31,32,33,34,35]. Elastin degradation is accompanied by elastases and elastolytic enzymes such as cathepsins and Mmp [25,33,38], we measured Mmp-9 mRNA expression in the aortic tissue, which significantly increased in CAM-treated tissue compared to its respective control. The respective elastin fragmentation may result in reduced breaking points found upon calcification induction.

Besides E_low_ providing information on elastin components, the stress-stretch stretch curve also delivers information regarding the collagen component of the vessel structure via the value of E_high_. Collagens are a family of 28 different subtypes, which are composed of specific triple helix structures. The production of mature collagen is a highly complicated process that is regulated by various biological molecules [39]. In the pathologic situation, disturbance in the balance of collagen synthesis and degradation ultimately drives diseases [39]. While collagen I shows pro-calcifying properties, collagen type IV and type XIV inhibit mineral deposition [39,40,41]. High expression of collagen I accelerates vascular calcification, while inhibition of collagen I can regress or slow calcification [39]. The collagen I expression is increased under high levels of phosphate, calcium, and uric acid [37]. These effects are linked with the osteochondrogenic trans-differentiation of VSMC [39,42]. As shown recently, inhibition of collagen type I by phosphate regulation-related drugs, such as Cinacalcet and Lanthanum, can ameliorate vascular calcification [43]. Collagen III is mainly found in extensible tissues such as blood vessels [44]. Mutations in the collagen III gene, as seen in the Vascular Ehlers-Danlos Syndrome (vEDS), lead to large vessel rupture and high morbidity and mortality in vEDS patients [45]. As we detected significant differences in E_high_ and the breaking points, we also measured collagen III mRNA expression in the aortic tissue. In addition, we detected a significant decrease in collagen III expression upon CAM stimulation compared to COM-stimulated tissue.

This proof-of-concept study indicates that, besides calcium quantification, the detection of biomechanical properties may further improve the analyzing tools for vessel calcification. Here, especially the vascular integrity as well as the influence of elastin/collagen determined by E_low_ and E_high_ on the pathophysiological function of the blood vessels could be further analyzed.

## 5. Conclusions

In conclusion, the determination of the biomechanical properties of a vessel using small-vessel myography represents a relevant extension of the analyzing tools in the research field of vascular calcification. Via stress–stretch curve measurements, the parameters investigated can potentially provide information on various aspects of vessel properties. In the presence of pronounced structural vessel abnormalities, ring fractures occurred at a lower stretch associated with a higher variation especially in the parts of the higher stretch. Therefore, E_low_ especially is of interest to discriminate for early changes in the elastic modulus. In terms of breaking points, λ appears to have a higher sensitivity to changes in the elastic modulus compared to σ, because at a lower elastic modulus the maximum stress required for the rupture of the ring is reached by a lower stretch. Thus, E_low_ and the breaking point λ could be valuable parameters for early detection of structural changes related to vascular calcification.

## Figures and Tables

**Table 1:** Comparison of biomechanical properties upon CAM treatment for 14 days compared to basal.

Parameter	Basal Mean	Basal SD	CAM Mean	CAM SD	T	*p*	d
E_low_	12.55	4.55	48.68	12.29	4.77	0.01	3.90
E_high_	3944.00	89.72	1439.27	90.92	33.96	<0.01	27.73
AUC	734.11	12.18	770.12	210.77	0.30	0.78	0.24
Breaking point (σ)	1884.74	41.54	1403.03	172.16	4.71	0.01	3.85
Breaking point (λ)	3.02	0.05	2.51	0.28	3.15	0.04	2.57
Calcium content (µg/mg)	3.13	3.50	249.15	152.96	2.79	0.05	2.27

CAM: calcifying medium, SD: standard deviation, *p*: *p* value, d: Cohen’s effect size.

## Data Availability

The data are available from the corresponding author upon reasonable request.

## Supplementary Materials

The following supporting information can be downloaded at: https://www.mdpi.com/article/10.3390/biomedicines11010211/s1, Figure S1: Effect of azathioprine on biomechanical properties under calcifying conditions; Table S1: Biomechanical properties basal (0 days); Table S2: Biomechanical properties upon 14 days of treatment with CAM.
