# Supplementary material for: Biomechanical Properties of the Aortic Wall: Changes during Vascular Calcification
Source: Biomedicines. 2023 Jan 14;11(1):211. doi: 10.3390/biomedicines11010211 (PMC9855732; doi:10.3390/biomedicines11010211)
Supplement: Supplementary file 1 [file biomedicines-11-00211-s001.zip › biomedicines-2127185-supplementary.pdf]

Supplementary Material

Biomechanical properties of the thoracic aortic wall: effect of location

Suppl. Table S1. Biomechanical properties basal (0 days). n=3 rats, each thoracic aorta into 18 rings.

Parameter	df1	df2	F	p	eta²	d†
E_{low}	17	34	0.9112	0.5679	0.3130	1.34
E_{high}	17	34	0.4810	0.9448	0.1939	0.98
AUC	17	34	0.7120	0.7695	0.2625	1.20
Breaking point (σ)	17	34	0.4203	0.9699	0.1736	0.92
Breaking point (λ)	17	34	0.6248	0.8483	0.2380	1.12
Calcium content(µg/mg)	17	34	1.0841	0.4059	0.3515	1.48

† d was calculated for better comparison with parameters in Table 3.
df1: count freedoms, df2: denominator freedoms, F: F test p: p value, eta^2: effect size, d: Cohen's effect size

Suppl. Table S2. Biomechanical properties upon 14 days of treatment with CAM.

Parameter	df1	df2	F	p	eta²	d†
E_{low}	17	34	0.5736	0.8882	0.2229	1.08
E_{high}	17	34	0.8865	0.5929	0.3071	1.34
AUC	17	34	0.8313	0.6494	0.2936	1.28
Breaking point (σ)	17	34	0.5911	0.8752	0.2281	1.08
Breaking point (λ)	17	34	0.6556	0.8217	0.2469	1.14
Calcium content(µg/mg)	17	34	0.9777	0.5025	0.3283	1.40

† d was calculated for better comparison with parameters in Table 3.
CAM: calcifying medium, df1: count freedoms, df2: denominator freedoms, F: F test p: p value, eta^2: effect size, d: Cohen's effect size

Method validation by a known inductor of vascular calcification: azathioprine

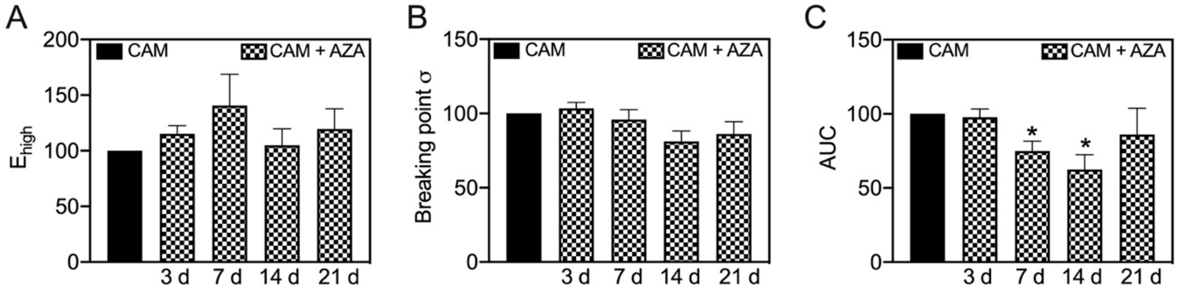

Suppl. Figure S1. Effect of azathioprine on biomechanical properties under calcifying conditions.
Aortic tissue rings were incubated for indicated time-points with calcifying medium (CAM) and azathioprine (100 µmol/l) and the stress-stretch curve and respective parameters were determined. Values are normalized to CAM stimulation at respective time-point. n=6, *p<0.05 compared to CAM at respective time-points. This Figure corresponds to Figure 7 of the main article.
